# Enhancing intrusion detection in encrypted DoH traffic through a robust ensemble learning framework

**DOI:** 10.1371/journal.pone.0345880

**Published:** 2026-04-07

**Authors:** Hussein Abrahim, Weiyan Hou, Yan Zhuang, Hafeez Ur Rahman

**Affiliations:** 1 School of Electrical & Information Engineering, Zhengzhou University, Zhengzhou, China; 2 School of Cyber Science and Engineering, Zhengzhou University, Zhengzhou, China; Cardiff Metropolitan University - Llandaff Campus: Cardiff Metropolitan University, UNITED KINGDOM OF GREAT BRITAIN AND NORTHERN IRELAND

## Abstract

The DNS over HTTPS (DoH) protocol enhances user privacy by encrypting DNS queries and responses within HTTPS. However, this encryption enables attackers to tunnel malicious traffic through covert DoH channels and make detection difficult for network security and enterprises. In this study, we propose a stacked ensemble approach that employs four sequential base learners’ — two Long Short-Term Memory (LSTM) and two Gated Recurrent Unit (GRU) neural networks as base classifier networks with varied hyperparameters and XGBoost (eXtreme Gradient Boosting) as the meta-learner. An XGBoost model trained on out-of-fold predictions generated by base learners supported through stratified 5-fold cross validation. We also applied Recursive Feature Elimination (RFE) with XGBoost for cross-validated feature selection and decreased high-dimensional feature spaces from 29 to 13 and 20 features on the CIRA-CIC-DoHBrw-2020 and DoH-Tunnel-Traffic-HKD datasets respectively. The proposed framework was evaluated on the CIRA-CIC-DoHBrw-2020 and DoH-Tunnel-Traffic-HKD datasets achieved 0.9996 recall, 0.9999 F1-score and 1.0000 AUC-PR on CIC-DoH dataset and 0.9998 recall, 0.9999 F1-score, and 1.0000 for both AUC-ROC and AUC-PR on HKD-DoH dataset. The proposed model outperforms all individual base classifiers, evaluated ML models, and existing state-of-the-art approaches. The proposed XGBoost meta-model needs only 1.26 seconds for training and 0.129 seconds for inference on HKD-DoH dataset, thus making the proposed framework both highly accurate and computationally efficient for real-world intrusion detection in encrypted DoH traffic.The preprocessed data and the source code supporting the findings of this study are available at: https://github.com/soreettihussein/Stacked-ensemble-meta-learning-IDS-for-DoH-network-traffic/tree/main.

## 1. Introduction

The Domain Name System, a fundamental component of the internet, has consistently drawn considerable interest. It simplifies the translation of human-friendly domain names into computer-recognizable Internet Protocol (IP) addresses. Because DNS is essential, security systems like firewalls usually do not block DNS packets. Consequently, attackers often use DNS as a hidden channel, known as DNS tunneling, to transmit stolen data. Data breach attacks may target both businesses and individual users [1–3]. These DNS vulnerabilities pose a major risk to internet users’ security.

Encrypting DNS traffic on the Internet is becoming more popular as a means of protecting privacy and securing networks. There have been numerous advancements in safeguarding DNS users’ privacy and security. One example is DNSSEC, which uses digital signatures and zone signing to preserve the integrity of DNS records so that end users can validate lookups [4–6]. DNS over TLS (DoT), DNS over QUIC (DoQ) and DNS over HTTPS (DoH) are more recent attempts to address user confidentiality and privacy in DNS data in transit [1,7]. In this study, we concentrate on DNS over HTTPS (DoH) as opposed to other techniques. DNS over HTTPS (DoH) was introduced by IETF in 2018 [8]. In recent years, the number of devices capable of supporting encrypted DNS has grown substantially. The recent versions of web browsers, such as Mozilla Firefox, Microsoft Edge and Google Chrome, now support DoH. In terms of OS, Windows 11 started supporting encrypted DNS networks using the DNS over HTTPS protocol. Launched in September 2020, macOS 11 and iOS 14 have a DoH network setup known as NDNSS Settings Manager [9].

The DNS over HTTPS protocol addresses the integrity and confidentiality issues of the Domain Name System protocol by encapsulating Domain Name System queries and responses within HTTPS. This enhances user privacy by preventing intermediary network devices between the client and DNS servers from seeing domain query activities. Additionally, the DoH protocol can help prevent man-in-the-middle attacks, eavesdropping, phishing attacks, and other similar threats [10–12]. However, encrypting DNS traffic also poses challenges for network administrators in managing network security services. Limited visibility into encrypted traffic diminishes the effectiveness of network security management and increases the risk of malware using encrypted DNS paths for communication. For example, on July 1, 2019, several Netlab security experts discovered and reported the first malware sample that utilized DoH. Another piece of malware, PsiXBot [12], was discovered in August 2019 and used DoH to add computers to its botnet and steal data. Additionally, Haddon et al. in [13] proposed a potential method of data exfiltration via DoH. Another instance of DoH abuse is when someone sends spam emails that contain harmful JavaScript loaded over DoH [14].

A number of previous studies have investigated supervised ML and DL approaches to address detection of malicious traffic in DoH networks. The most widely used algorithms are RF, DT, KNN, Naive Bayes, Gradient Boosting, SVM, and Logistic Regression [15,16]. For example Moure Garrido et al. [13] applied DT and RF to statistical analysis on the CIRA-CIC-DoHBrw-2020 dataset and their study achieved a detection rate above 95%. Ali and Chen [17] proposed artificial artificial intelligence-driven intrusion detection framework for DoH networks. Their proposed system combines Capsule Networks (CapsNets) with Graph Transformers and Contrastive Self-Supervised Learning (CSSL) to identify malicious attacks hidden within encrypted DoH traffic, such as polymorphic malware, DNS tunneling, and TLS session hijacking. while Aggarwal and Kumar [18] proposed an ensemble framework combining multiple ML classifiers for DoH detection.

Deep learning techniques have been demonstrated promising results in capturing temporal and sequential patterns in encrypted network traffic. Study in [19] applied LSTM architectures for DNS tunnel detection without manual feature engineering. Zuo et al. [1], proposed METC, a hybrid CNN-BiGRU framework with multi-head attention for cross-network encrypted DoH traffic detection. Jung and Kwak [2], proposed MTL-DoHTA, a multi-task learning framework using 2D-CNN with GradNorm and attention mechanisms. Their approach achieved a macro-averaging F1-score of 0.9905 on the CIRA-CIC-DoHBrw-2020. Furthermore, the study in [20] presented hybrid CNN-LSTM models for DNS tunnel attack detection, illustrating the effectiveness of integrating spatial and temporal feature learning. A survey presented by Lyu et al. [21] studied DNS encryption techniques, malware misuse patterns and inference methods highlighting the need for more robust detection frameworks.

Regarding ensemble learning for intrusion detection, stacking-based methods have gained significant traction in recent years. Amara et al. [22] proposed a stacked ensemble of DL models (CNN, LSTM, and GRU) for robust intrusion detection in IoT network environments. Similarly, a stacking ensemble for IoMT environments presented [23] combines ML and DL classifiers and demonstrates superior detection accuracy compared to individual models. In the broader network security domain, ensemble stacking learning has been shown to enhance intrusion detection.

Although research on detection of malicious traffic in DoH network has expanded in recent years there are still limitations in the reliability of current systems. Most works rely on individual classifiers such as RF [3,15],CNN [12], or single LSTM [24] which struggle with the high-dimensional, non-linear feature spaces of encrypted DoH traffic, where behavioral signatures of tunneling tools (dnscat2,tuns, dnstt, dns2tcp, tcp-over-dns, and iodine) overlap heavily with benign traffic. LSTM and GRU networks remain underexplored for DoH detection and classification and the few studies that use them deploy a single model without leveraging diversity from combining multiple sequential networks. Notably, despite the proven benefits of both sequential deep learning and ensemble stacking in other security domains, [25–28], no previous work has combined stacked ensemble learning with LSTM and GRU models specifically for malicious DoH tunnel detection. The class imbalance in DoH datasets is also widely neglected, many studies [29–31] ignore this issue, while others [32] use SMOTE which can degrade generalization. Additionally, a substantial portion of previous studies [1–3,33] relies on single train-test splits without cross-validation and skips rigorous feature selection [33,34] increasing the risk of overfitting. To address these limitation, we proposed a stacked ensemble by combining two LSTM and two GRU under an XGBoost meta learner, with stratified 5-fold cross-validation for imbalance handling, cross-validation RFE and XGBoost for feature selection. We evaluated performance of proposed framework on two datasets. The contributions of our studies are summarized as follows

- We introduced and implemented a method for detecting malicious DoH traffic by employing GRU and LSTM as base classifiers, with eXtreme gradient Boosting as a meta-learner in a stacking ensemble model. Instead of depending only on the predictions of base classifiers, the meta-learner is trained on a set of meta-features derived from out-of-fold predictions, which helps minimize prediction bias and strengthens both the reliability and objectivity of the final ensemble decision..
- Our proposed method incorporates k-fold cross-validation, hyperparameter optimization, stratified sampling, and feature selection to address issues related to bias, overfitting, and imbalanced datasets.
- We evaluate the proposed stacked ensemble framework against recent methods for malicious DoH traffic detection reported in the literature. Model performance is measured through six metrics: accuracy, precision, recall, F1-score, AUC-ROC, and AUC-PR.

The remainder of this paper is structured as follows. Section 2 presents reviews of related works. Section 3 offers a detailed introduction to the proposed model and its algorithm. Section 4 presents experimental results and discussions, including evaluation metrics, and analyzes the outcomes of our experiments on a real dataset. Finally, Section 5 concludes the paper and outlines directions for future work.

## 2. Related works

Detecting DoH protocol tunnels has become a challenging task for traditional DNS tunnel detection tools. Researchers have been focusing on developing detection methods, especially for DoH traffic, in recent years [35].

The work presented in [34] constructed an autoencoder network using Bi-LSTM and proposed an autoencoder-based DoH traffic anomaly detection system. The study proposed by [33] also utilized autoencoders to distinguish between malicious DNS over HTTPS traffic and benign DoH traffic. Additionally, the researchers in [31] suggested a Transformer-based system for detecting DoH attacks to differentiate between malicious and benign DoH traffic. Furthermore, the CIRA-CIC DoHBrw-2020 dataset was examined by the authors of [30] utilizing multi-head attention and residual neural networks. Additionally, they developed a thorough DNS over HTTPS detection system that can be deployed in an enterprise network to enhance the security of the system. The work in [36] proposed an intrusion detection system based on Explainable AI to detect malicious activity in DoH networks to support network administrators in securing their networks. Their IDS system was developed using balanced and stacked RF. The study in [32] proposed a DoH tunneling detection system that uses Ant Colony Optimization (ACO) for feature selection on the CIRA-CIC-DoHBrw-2020 dataset. ACO reduced the original 29 statistical features down to 15 for both multiclass and binary classification. The selected features were then fed into XGBoost, KNN, Random Forest, and CNN classifiers, with hyperparameters tuned using Gaussian optimization and class imbalance handled through SMOTE. Their approach achieved 99.99% accuracy in binary classification and 99.55% in multiclass classification, the authors did not disclose which features were selected and used for the classification tasks.

The authors in [11] introduced a dual-layer detection strategy to differentiate between malicious and benign DNS over HTTPS traffic, as well as to separate non-DoH from DoH traffic within DNS over HTTPS traffic. Their research employed time series features to examine packet flows. They confirmed the utility of time features for detecting DNS over HTTPS tunnels and contributed a dataset named CIRA-CIC-DoHBrw-2020, which has recently gained widespread use in research. In [9], the authors proposed an ML approach for classifying malicious Domain Name System tunnel tools through DoH traffic analysis. This method relies on hierarchical ML categorization and focuses exclusively on DNS over HTTPS traffic investigation. The evaluation results indicate that their approach achieves a 98.02% classification accuracy and can detect six malicious DNS tunnel tools (dns2tcp, iodine, dnscat2, tuns, dnstt, and tcp-over-DNS), although it only identifies tunneling attacks. To avoid unauthorized data extraction, the authors of [37] proposed an architecture designed to examine and identify malicious DoH communications. The results reveal that DoHxP precisely detects malicious traffic with 99.78% accuracy and a misclassification rate of only 0.22%. DoHxP correctly classifies 99.22% of benign traffic as benign, with a 0.78% misclassification rate as malicious.

The other works in [38] proposed a black-box attack framework for evading ML-based DoH tunnel detection systems, built around their Strategic Feature-Adaptive Adversarial Attack (SFAA) algorithm that leverages feature correlations and SHAP-based importance scores to craft realistic adversarial traffic samples. They trained a surrogate MLP model, generated adversarial samples, filtered unrealistic ones via Mahalanobis distance, and transferred attacks to target classifiers, achieving a 63.26% attack success rate across seven ML models. The study also proposed the Dual-Defense Adversarial Framework combining adversarial training with confidence-driven secondary classification to restore model robustness. Their findings demonstrate that even high-accuracy DoH tunnel detectors remain vulnerable to carefully crafted adversarial perturbations under practical black-box settings.

Studies presented in [10] proposed the detection of unauthorized data extraction over DNS over HTTPS tunnel using three machine learning algorithms, namely Random Forest, Boosted Decision Tree and Logistic Regression (LR). The authors generated a custom dataset and extracted flow-based features, mainly TLS record length and time interval features. They also computed the maximum, minimum, mean, and standard deviation for all flow-level features. The experimental results show that the proposed method can detect DNS over HTTPS tunnels with 99% accuracy. Furthermore, they analyzed the impact of various server locations on the detection of DoH tunnels. In another study, they proposed hybrid deep learning methods for identifying and classifying malicious use of DNS over HTTPS network traffic [1]. The authors developed an integration of CNNs and BiGRUs to enhance the detection of malicious tunnels in DoH for both mobile networks and desktop networks.

The study presented in [39] introduced WEDoHTool, a method for early identification of specific DoH tunnel tool traffic in dynamic network environments using word embedding technology. Instead of relying on statistical flow features that require complete flows or time windows, the study extracts TLS record length sequences from just the first few packets of each unidirectional flow and transforms them into embedding vectors using word2vec. A two-stage classification approach first filters DoH traffic from background HTTPS using a lightweight TextCNN, followed by a Transformer encoder that identifies the specific tunnel tool (dns2tcp, dnscat2, iodine, dnstt, tcp-over-dns, or tuns). The authors evaluated their proposed method on the combined CIRA-CIC-DoHBrw-2020 and DoH-Tunnel-Traffic-HKD datasets, WEDoHTool maintained at least 98.82% and 98.07% accuracy across both stages even under simulated packet loss conditions. The authors of [40] proposed detecting malicious usage of the DoH traffic system utilizing statistical analysis. They analyzed variations observed between malicious and benign traffic using statistical analysis. The statistical features that the authors utilized are the time between two packets, packet size, and connection duration. These features are extracted from the PCAP of the CIRA-CIC-DoHBrw-2020 dataset. The result of the experiment shows that the proposed system's detection rate exceeds 95% for malicious connections.

Despite significant progress in detection and classification of DoH network traffic, existing studies still show limitations in this domain. Many approaches rely on single classifiers unable to capture the full range of patterns in encrypted DoH traffic, evaluate models on single source dataset, do not address class imbalance properly and overfitting issues through stratified cross-validation. Additionally, feature selection and robust ensemble approaches combining sequential deep learning algorithms remain insufficiently explored. These limitations highlight the need for a generalized and bias-resistant DoH malicious traffic detection framework, motivating the development of the proposed stacked ensemble that integrates LSTM and GRU as base learner with XGBoost as meta learner. Table 1 presents a summary of related works.

**Table 1:** Summary of related work on malicious DNS over HTTPS traffic detection.

Ref	Year	Dataset	Method	Contribution	Evaluation Metrics	Metric value	Limitation
[1]	2025	DoHBrw-2020, DoH-DGA-HKD DoH	CNN-Bi-GRU	Benign DoH vs. malicious DoH traffic	Accuracy, Precision, recall, F1-score	97.36, 97.36, 97.36 97.34	Did not tackle the imbalanced data issues
[2]	2025	DoHBrw-2020, DoH-Tunnel-HKD	2DCNN+ Gard Norm +Attention	DoH vs. non-DoH, benign vs. malicious	Macro-avg-F1-score	0.9988	No feature selection and crossvalidation is not implemented
[38]	2025	DoHBrw-2020	DDAF + RF	Classify benign vs. malicious	Accuracy, Precision, recall, F1-score	99.45, 0.9947, 0.9920, 0.9934	Single source data, cross validation, is not implemented,
[39]	2025	DoHBrw-2020, DoHTunnel-HKD	lightweight TextCNN, Transformer-encoder	Classify HTTPS/DoH/ malicious DoH, tunneling tools	Accuracy, Precision, recall, F1-score	99.66%, 99.30%, 99.31%, 99.30%	Cross validation, is not implemented. Did not tackle the imbalanced data issues
[32]	2025	DoHBrw-2020	XGBoost, KNN, RF, CNN	DoH vs. non-DoH, benign vs. malicious	Accuracy, Precision, recall, F1-score	0.998, 1.000, 0.999, 1.000	Cross validation not implemented and single source data,
[7]	2024	DoHBrw-2020	CGAN	Classify benign vs. malicious	Accuracy, Precision, F1-score, recall, ROC-AUC, AUC-PR	0.9974, 0.9978, 0.9977, 0.9976, 0.9985, 0.9996	Single source data, cross validation, is not implemented, no feature selection
[3]	2024	DoHBrw2020, DoH-Tunnel-Traffic-HKD,Czech Republic	RF	Classify benign vs. malicious	Precision, recall, Accuracy, F1-score	0.973, 0.983, 0.958, 0.970	k-cross validation is not used and did not handle data imbalanced.
[33]	2023	Custom	Auto-Encoder	Classify DoH vs malicious	Accuracy, F1-score, Precision, recall, AUC,	0.98874, 0.99831, 0.98756, 0.99264, 0.99384	Feature selection not considered, Single source data
[9]	2023	DoH-Tunnel-Traffic-HKD, DoHBrw-2020	XGBoost, Light GBM, CatBoost	HTTPS/DoH/ malicious DoH, tunneling tools	Accuracy, Precision, recall, F1-score	0.999, 0.999 0.999, 0.999	Not enough performance metrics, no hyperparameter optimization
[15]	2023	DoHBrw2020, DoH- Tunnel-Traffic-HKD	RF, DT, GB, KNN	Classify HTTPS/DoH/ malicious DoH	Accuracy, Precision, recall, F1-score	0.993, 0.993, 0.994, 0.993	Did not tackle the imbalanced data,cross validation not implemented
[36]	2022	DoHBrw-2020	RF, Stacked RF	Classify HTTPS/DoH/ malicious DoH	Accuracy, F1-score, Precision, recall	0.9998, 0.9991 0.9991, 0.9992	Balance data with SMOTE, Single Source of data
[34]	2022	DoHBrw-2020	Auto Encoder, LSTM, Bi-LSTM	Detect benign vs. malicious	Accuracy, Precision, Recall, F1-score	99.6%, 98.67%, 97.53%, 99.7%	Feature selection not implemented and single source data
[31]	2022	DoHBrw-2020	RF, MLP, C4.5, GB, SSSVM	Detect and classify DoH/ malicious DoH	Precision, Recall, F1-score	0.993, 0.993, 0.993	Did not tackle the imbalanced data,Single source data
[10]	2021	DoHBrw-2020	1D CNN-4, 1D CNN-5, 2D CNN-6	Classify DoH/ malicious DoH	Accuracy, F1-score, Precision, recall	0.9841, 0.9884, 0.9765, 0.9841	Feature selection not implemented, single source data
[11]	2020	DoHBrw-2020	RF, KNN, 2D CNN, C4.5	Classify HTTPS/ DoH/ malicious DoH	Precision, recall, F1-score	0.999, 0.999, 0.999	Single source data; no cross-validation,
**Ours**		**DoHBrw-2020, DoH-Tunnel-Traffic-** **HKD**	**Proposed** **Stacked** **Ensemble** **(LSTM+** **GRU+** **XGBoost)**	**Classify DoH/** **malicious DoH**	**Accuracy, Precision, recall,** **F1-score ROC&PR,**	**0.9998,** **1.0000, 0.9998,** **0.9999, 1.0000,** **1.0000**	**Considered all above-mentioned limitation**

## 3. Proposed system

**Fig 1:**
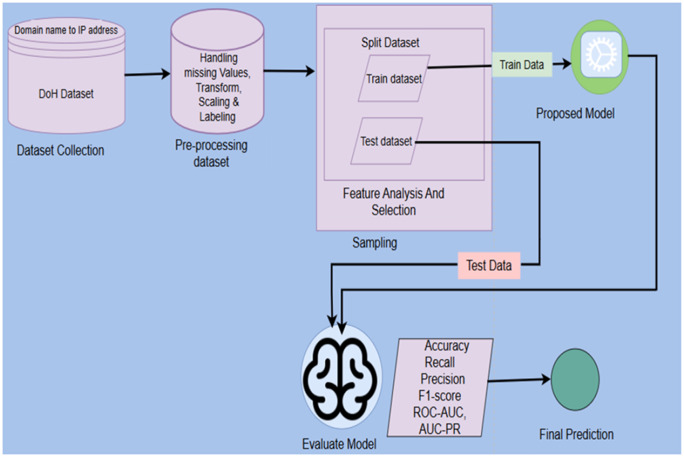
Architecture of Proposed System for DoH tunnel malicious Detection.

Malicious activity detection in DoH tunneling involves modeling network flows and learning to classify malicious activity. The goal of our studies is to differentiate malicious DoH tunneling traffic from benign DNS over HTTPS traffic. This paper introduces a stacked sequential ensemble model that precisely detects malicious tunnel traffic, thereby preventing security issues arising from DoH tunneling. In this study, we employ a stacked ensemble learning method to categorize DoH traffic as either benign or malicious. The overall proposed architecture for malicious DoH tunneling detection is illustrated in Fig 1. The proposed framework operates through five sequential steps that form an end-to-end system for detecting malicious use of DoH networks. In thefirst step (Section 3.1), we present dataset preparation and preprocessed CIC-DoH and HKD-DoH datasets by removing null values, encoding the target labels and applying StandardScaler. In the second stage (Section 3.2), we performed feature analysis using Kernel Density Estimation (KDE) plots to visualize the importance of each feature across both datasets. At the third stage (Section 3.3), we applied feature selection using Recursive Feature Elimination with XGBoost, then the feature space was reduced from 29 to 13 for CIC-DoH and from 29 to 20 for HKD-DoH datasets. In the fourth step (Section3.4), we present the core framework where four diverse sequential base learners (LSTM1, GRU1, LSTM2, GRU2) are each trained on K-1 folds and generate out-of-fold prediction scores, which are stacked into a meta-feature matrix and used to train the XGBoost meta-learner. In the final step (Section 4), the fully trained base models generate predictions on the unseen test set, these are stacked and passed to the meta-learner for final classification.

### 3.1. Dataset description

In our study, we utilized two real-time datasets: the CIRA-CIC-DoHBrw2020 [11] and the DoH-Tunnel-Traffic-HKD dataset [9]. The CIRA-CIC-DoHBrw-2020 dataset was provided by CIC (Canadian Institute for Cybersecurity) and includes benign and malicious DNS over HTTPS traffic along with non-DoH traffic. Dataset traffic was generated by visiting the top 10k websites using browsers that support the DoH protocol, such as Google Chrome and Mozilla Firefox. Additionally, the attacker’s traffic was generated using DNS tunneling tools: dns2tcp, dnscat2, and iodine. DNS over HTTPS traffic between the DoH proxy and the DoH server was captured. AdGuard, Quad9, Cloudflare, and Google are the DoH servers that are utilized. The DoH-Tunnel Traffic-HKD dataset holds data traffic from three newly emerging DoH tunneling tools: tuns, tcp-over-dns, and dnstt. The data was generated and collected over 48 hours, and then, it was assumed that 20 clients utilized the tools.The dataset we used to evaluate the proposed method contains 35 features. However, we removed network environment specific features such as Source IP, Destination IP, Source Port, Destination Port and Time Stamp [9]. In this research, we focused solely on differentiating malicious and benign DoH traffic, and our proposed model was evaluated on both datasets. Both datasets are provided as CSV files, and our study operates on pre-computed flow-level statistical features (provided as CSV files), not on raw packet payloads or image representations of network traffic. The features used in this study were originally extracted from PCAP captures using the DoHMeter tool [11] by dataset providers, which computes statistical features per network flow based on packet length statistics, timing statistics, flow volume, and response time characteristics.

The selection of these two datasets was based on three main reasons. First, the CIRA-CIC-DoHBrw-2020 [11] and DoH-Tunnel-Traffic-HKD [9] datasets are the most widely adopted public benchmarks specifically designed for malicious DoH tunnel traffic detection, enabling direct comparison with existing studies. Second, the two datasets provide complementary attack coverage: the CIC dataset contains traffic from well-established tunneling tools (dns2tcp, dnscat2, iodine), while the HKD dataset includes traffic from newly emerging tools (tuns, tcp-over-dns, dnstt). Evaluating on both allows us to assess our model’s generalizability across different generations of attack tools addressing the single source of data limitation identified in many prior studies (Table 1). Third, both datasets share the same 29 statistical features and the same benign traffic baseline, enabling controlled cross-dataset analysis where performance differences can be attributed to variations in attack tool behavior rather than confounding factors.

#### 3.1.2. Preprocessing dataset.

To develop a better model that achieves high performance, it is necessary to examine the dataset's integrity. In both our datasets, we removed all missing values or null attributes from the training set and test set data. Additionally, we transformed all features of the dataset into a numerical form for computation to be performed on it. Since they contain sophisticated mathematical processing, we can’t input non-numeric values into models [27]. Furthermore, the target column is encoded where “0” represents benign traffic, while “1” indicates malicious traffic. Since the features of the dataset are scaled at different values, which leads some features to have larger values, we used feature normalization based on the StandardScalar to standardize the data. This transformation standardizes the data by setting the mean to 0 and the variance to 1, using the standard normal distribution (SND).


$$\text{ z}=\frac{\text{x }-\;\mu }{\text{sd }}$$ (1)

Here, x denotes the original feature value, μ is the feature’s mean, and sd refers to its standard deviation. In addition, we have imbalanced data in both our datasets, CIC and HKD. As presented in Table 2, the CIC dataset contains 269643 records, which means the proportion of malicious traffic and benign traffic is 249836:19807, indicating an imbalanced dataset. Additionally, the HKD dataset contains 124967, as presented in Table 3. The ratio between malicious traffic and benign traffic is 105160:19807, indicating an imbalance in the data. Benign traffic is the same for both our datasets because the standardized DoH protocol generates it. To tackle the problem of data imbalance in DoH traffic, we employed stratified sampling techniques to manage an uneven dataset, and overfitting was mitigated by the k-fold cross-validation method. We have a biased class (imbalance); it is important to ensure that the amount of benign traffic is nearly equal across all segments. For this purpose, we utilize a stratified k-fold cross-validation technique, which is akin to cross-validation with k subsets but generates a stratified sample as an alternative of a random one. This method divides the dataset so that all class data are evenly distributed between the training and test sets, reflecting the entire dataset in proportional amounts. After applying this approach, the training dataset is split into 5 folds, trained using 𝑘 − 1 folds, and the remaining 1 fold is used to validate the model. The outputs of these base classifiers are then used as meta-features for a meta-learner (stacked ensemble model)

**Table 2:** Shows the CIC dataset.

Total traffic	Malicious	Benign traffic	Class
269643	249836	19807	2

**Table 3:** Details of HKD dataset.

Total traffic	Malicious	Benign traffic	Class
124967	105160	19807	2

### 3.2. Feature analysis

We performed feature analysis to learn which feature can differentiate malicious and benign traffic in network flow of DoH traffic. As we mentioned in section 3.1, we used features of CIC and HKD dataset. The feature lists for both datasets are the same, however they differ in the tunneling tools used by authors or providers to generate malicious DoH traffic. In HKD dataset malicious DoH traffic generated by newly emerging tunneling tools while malicious DoH of CIC dataset generated by well-known DNS tunneling tool. The features used for analysis in this study are described in [9,11]. Feature analysis in machine learning (sequential model) is crucial for understanding features significance. The main of the feature examination is to well recognize the data. It is important for the understanding the differences between malicious DNS over HTTPS and benign DoH traffic.

Our feature analysis approach was performed using Kernel Density Estimation (KDE) plot. A Kernel Density Estimation (KDE) plot which was first developed by Silverman [41] resembles a histogram except that it displays the estimation of a variable's probability density function rather than discrete bins. A histogram displays the frequency/count, whereas a Kernel Density Estimation plot displays the density on the y-axis. Each feature's value distribution is displayed using a Kernel Density Estimation plot. Histograms may also be used, but this would require figuring out how many bins would best represent the distribution of the data, and because there are so many features for two datasets, manual modifications take a lot of effort. The KDE plot also has the benefit of providing a line rather than bars, which facilitates the comparison of several classes on a single graph. Feature distributions of the most interesting features were plotted using KDE plot. Due to limitation of space, we present some of interesting feature value distribution and discussed them.

**Fig 2:**
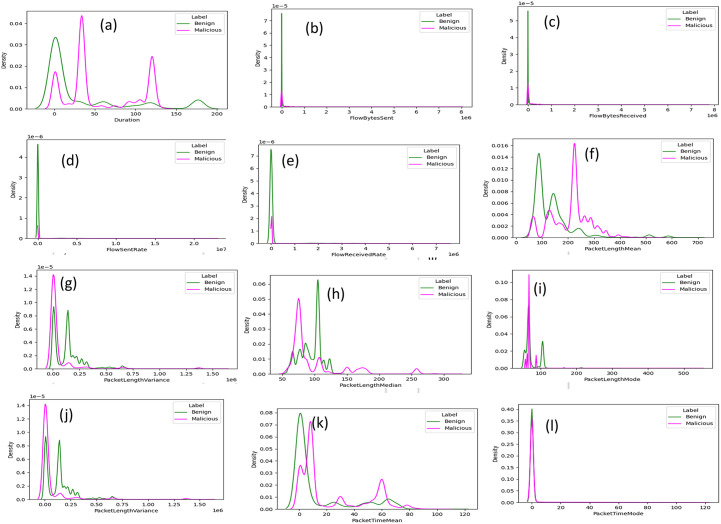
Distributions of features on CIC dataset.

**Fig 3:**
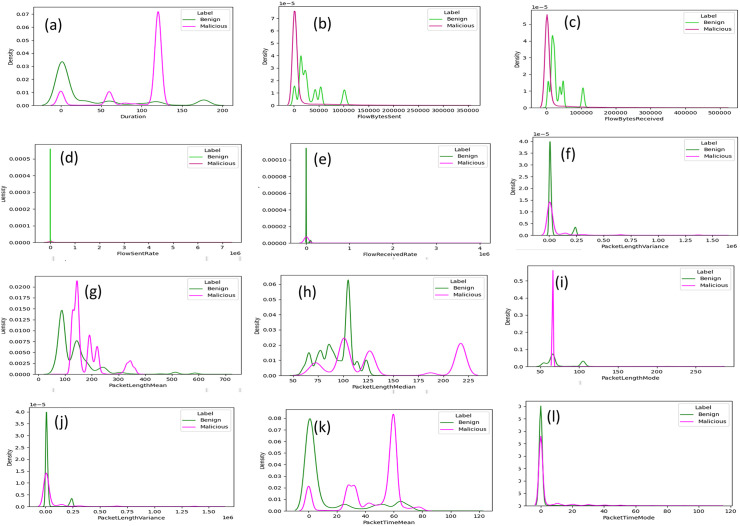
Distributions of features on HKD dataset.

Fig 2(a) shows value distribution of duration features of CIC dataset, duration of traffic flows shows clearly distinguishing malicious from benign DoH traffic. Malicious DoH traffic has a shorter duration than benign DoH traffic. The same hold for duration feature of HKD dataset, as shown in Fig 3(a). However, distribution of duration feature of malicious and benign overlap at the beginning of transmission of flows but later separated.

Flow byte sent feature of CIC dataset can clearly distinguish malicious and benign traffic as presented in Fig 2(b). Malicious DoH traffic flow bytes sent is higher than benign DoH traffic sent bytes. There is only small portion of overlap in this feature value distribution. Also, in HKD dataset number of bytes sent provides helpful patterns to differentiate malicious from benign traffic represented in Fig 3(b). Flow byte received has the same proportional to sent bytes in both datasets as shown in Fig 2(c) and Fig 3(c) respectively for CIC and HKD dataset. Rate of flow sent of malicious and benign DoH traffic of CIC dataset shown in Fig 2(d). Flow sent rate of malicious traffic is higher than flow sent rate of benign DoH traffic. It is interesting there is no overlap that leads to confusion to distinguishing of malicious and benign traffic. For HKD dataset distribution of flow sent rate feature found in Fig 3(d). In addition, flow received rate of CIC dataset for both malicious and benign traffic illustrated on Fig 2(e) with clearly boundary between two classes. flow received rate of HKD dataset presented in Fig 3(e). A bytes sent and received by malicious DNS over HTTPS rises logically due to tunneling techniques, which are designed to transfer additional data, are employed.

Like the flow bytes delivered and accepted, packet length mean indicates that larger packets are used in tunneling. Fig 2(f) illustrate the typical packet lengths of malicious DoH flows are obviously longer than those of benign DoH flows correspond to CIC dataset. This is the same for HKD dataset packet length mean as demonstrated in Fig 3(f). Packet length variance is another aspect associated with packet length. Benign DoH traffic has a lower packet length variance than malicious DoH traffic. Because in benign DoH traffic, packet sizes are relatively constant because DNS queries and responses relatively have uniform sizes and predictable. For both CIC and HKD dataset variance of packet length shown in Fig 2(g) and Fig 3(g) respectively, which almost perfectly distinguishes benign from malicious DoH traffic. The other most important packet length statistics features are mode and median value distribution. Benign DoH has a lower mode and median value distribution compared to malicious DoH. Since a flow with a lower packet length, will also have lower median and mode value distribution this holds true for CIC and HKD dataset as presented in Fig 2(h,i),Fig 3(h,i) in sequence orders.

The Canadian Institute for Cybersecurity dataset comprises a packet time variance feature, which nearly perfectly differentiates malicious DoH from the benign traffic classes as shown in Fig 2(j). The difference in the packets’ inter-arrival times, or packet time variance, is greater in benign DoH traffic than in malicious DoH traffic. Packet Time Variance value distribution demonstrates the same behavior in HKD dataset as in CIC dataset as represented in Fig 3(j). This is similar to what one would expect based on the distribution of duration feature. The mean packet time of HKD dataset clearly shows border between malicious traffic and benign traffic and mean packet time of benign traffic is greater than malicious traffic. However, packet time mean of CIC dataset shows slight overlaps between both classes, but still it shows difference between benign and malicious at some points figure represented in order their discussion in Fig 3(k) and Fig 2(k). Malicious DoH traffic has larger Packet Time Mode than benign traffic in both HKD and CIC dataset as illustrated in Fig 3(l) and Fig 2(l) respectively with almost perfect to distinguish both classes.

It is possible to determine which features are more and less important for the detection malicious use of DoH by analyzing each feature for both datasets. Features with less overlap are very important for distinguishing malicious DoH traffic from benign DoH traffic.

Generally, in the CIC dataset, packet time mean, packet length median, duration, flow bytes received and packet length mode are the most significant features for determining attackers’ activity in DoH networks. Most important features for malicious action detection in DoH networks in the HKD dataset are, packet time skew from mode, flow bytes received, packet length mode, packet length median, packet length variance, and packet length mode. These are meant to capture both timing pattern and statistical aspects of network flow that are critical in identifying DoH-based attack-related abnormalities.

This is also as indicated by the feature selection findings in Section 3.3, presenting the consistency and relevance of these features with different datasets. By identifying the most informative features, the detection models can be enhanced in performance with reduced computational complexity. The analysis confirms that time-related and statistical packet flow features are significant indicators in the identification of malicious DoH traffic.

### 3.3. Feature selection

**Fig 4:**
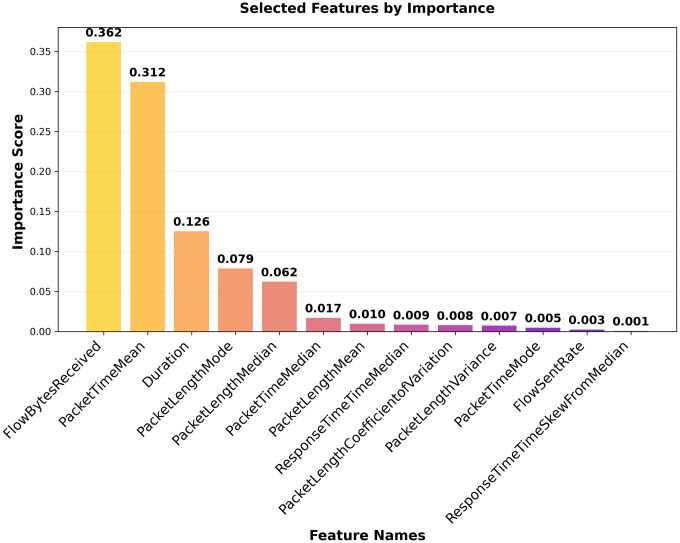
CIC dataset feature Importance.

**Fig 5:**
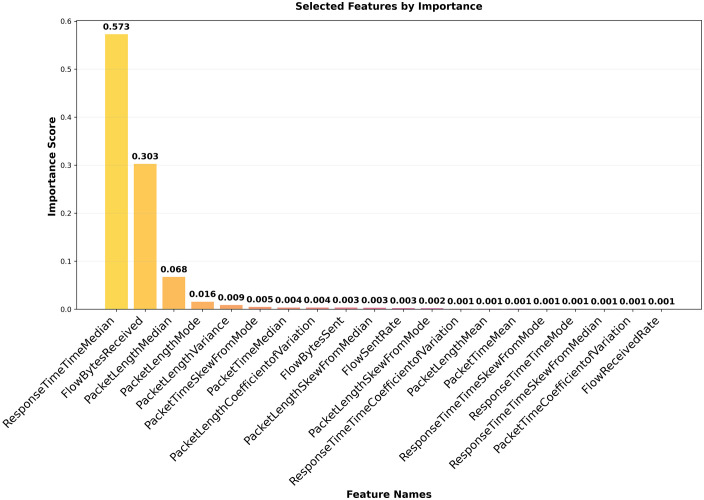
HKD dataset feature importance.

Besides dataset balancing, feature selection is also important for optimizing the performance of a classification model. The objective is to select the smallest feature subset that still enables accurate classification. In this work, we used a feature selection approach based on Recursive Feature Elimination (RFE) [42] and the XGBoost model. First, every feature is chosen during the RFE process. Using a feature-significant ranking, the least significant feature is eliminated from the feature set. The most suitable feature count can be found by repeatedly pruning features and measuring their impact on model performance. The features should be able to be ranked in order of importance by the ML classification model used for Recursive Feature Elimination. XGBoost is a decision tree-based model that can demonstrate the importance of each feature and is suitable for RFE. The feature selection algorithm, based on RFE and XGBoost, is shown in Algorithm 1. Upon applying the feature selection algorithm, the number of features for the CIC dataset was reduced from 29 to 13, as demonstrated in Fig 4, and from 29 to 20 for the HKD datasets, as shown in Fig 5. Thus, selected features were utilized for the proposed model to distinguish malicious from benign traffic.

**Algorithm 1:** **Feature Selection**

**Input:** Dataset X, target y, feature count to select k

**Output:** Selected features:

**1**. Partition the data (X and y) into training and testing subsets: (X_train, y_train) and (X_test, y_test)

**2**. Initialize XGBoost classifier: model

**3**. Initialize RFE:

Set RFE with estimator = model

Set n_features_to_select = k

**4**. Train RFE on training data:

RFE.fit (X_train, y_train)

**5**. Extract selected features:

selected_features = indices or names where RFE.support_ = True

**6**. Decrease datasets to selected features:

X_train_rfe = X_train[selected_features]

X_test_rfe = X_test[selected_features]

**7.** Train proposed model:

model.fit (X_train_rfe, y_train)

### 3.4. Proposed model

Our proposed method has two layers. The first layer consists of a number of GRU and LSTM units, which are referred to as the base classifier, and the second is an XGBoost classifier, which is called the meta learner. It is used to train on the output prediction of the first layer using a stacking mechanism. In this section, both GRU and LSTM, as well as XGBoost and stacked ensemble learning models, are explained.

#### 3.4.1. LSTM.

**Fig 6:**
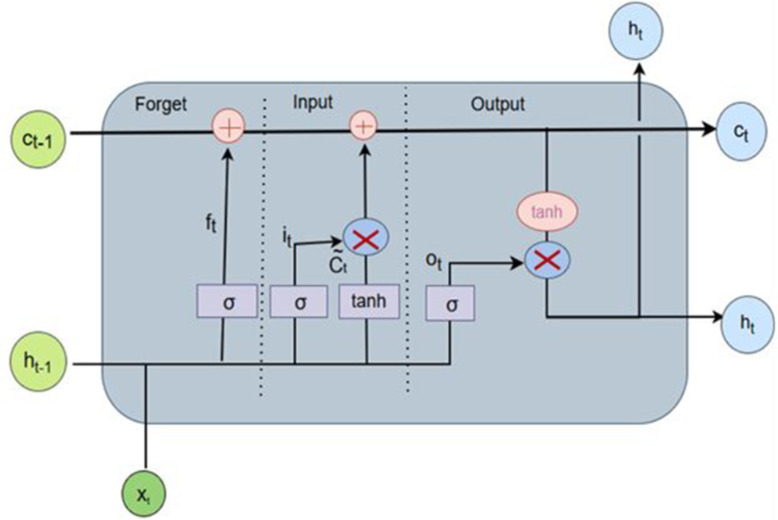
Structure of the LSTM.


LSTM is an enhanced version of RNN, with the ability to learn long-term dependencies. In addition, LSTM overcomes the vanishing and exploding gradient problem that simple RNNs face. This makes it suitable for detecting sequential data, such as DoH traffic flows. Long Short Term Memory comprises a memory cell c_t_, an input gate i_t,_ a _forget gate_ f_t,_ and an output gate o_t_ [43,44]. The state from the previous state is stored in the memory cell. The input gate decides how much of the input data should be retained in the unit state at the current time step, t. The forget gate f_t_ determines whether information from the previous time step (t − 1) should be discarded or passed to the input gate as retained information. The output gate o_t_ decides which information will be used as the output. Fig 6 illustrates the structure of the LSTM used in this study. The subsequent mathematical formulas explain how information moves through the LSTM layers:

$${\text{f}}_{\text{t}}=\sigma ({\text{u}}_{\text{f}}{\text{x}}_{\text{t}}+{\text{w}}_{\text{f}}{\text{h}}_{\text{t}-\text{1}}+{\text{b}}_{\text{f}})$$ (2)

Once a decision to keep the data has been made, the cell state is updated by an input gate i_t_


$$\;{\text{i}}_{\text{t}}=\sigma \left({\text{u}}_{\text{i}}{\text{x}}_{\text{t}}+{\text{w}}_{\text{i}}{\text{h}}_{\text{t}-\text{1}}+{\text{b}}_{\text{i}}\right).$$ (3)

The hyperbolic tangent function then produces a vector of new potential values *𝑐*_t_.


$$\overset{\sim }{\underset{t}{c}}=\text{tanh}\left(u_{c}x_{t}+w_{c}h_{t-\text{1}}+b_{c}\right).$$ (4)

The current candidate value is then multiplied by the previous one through the use of f_t_ to determine what should change. *i*_*t* *_𝑐_*t*_ is also introduced into the equation


$${\text{c}}_{\text{t}}={\text{c}}_{\text{t}-\text{1}}⊗{\text{f}}_{\text{t}}+\overset{\sim }{\underset{t}{c}}⊗{\text{i}}_{\text{t}}.$$ (5)

Lastly, the last output is a filtered output *𝑜*_*t*_ of the cell state


$${\text{o}}_{\text{t}}=\sigma \left({\text{u}}_{\text{o}}{\text{x}}_{\text{t}}+{\text{w}}_{\text{o}}{\text{h}}_{\text{t}-\text{1}}+{\text{b}}_{\text{o}}\right).$$ (6)

$${\text{h}}_{\text{t}}={\text{o}}_{\text{t}}⊗\text{tanh}\left({\text{c}}_{\text{t}}\right).$$ (7)

where o_t_ output gate, i_t_, input gate, f_t_ forget gate and c_t_ memory cell and C_t,_ C_t_^∼^ and C_t−1,_ represents, the current time step, the candidate cell state, and the unit memory (cell state) at the previous time step respectively. Also, W_i,_ W_f,_ W_o,_ and W_c_ are weight matrices. In addition, where b_*_ is the bias and the weight matrix is U*, which attaches the inputs to the current hidden layer. The tanh and σ are the activation functions of tanh and sigmoid, respectively. This “⊗” refers to a component-wise operation. Finally, the output h_t_ is determined by the output gate and the information stored in the memory cell.

#### 3.4.2. GRU.

GRU, introduced by Cho et al. [45], has a working mechanism similar to the Long Short-Term Memory, but without an output gate. The update gate (z_t_) and reset gate (r_t_) are the two gates that the GRU substitutes for the three in the LSTM. The reset gate regulates the information removed from memory, while the update gate controls the information added to memory.

These gates are essentially vectors that control the data sent to the output. They can be taught to retain previously collected data or to exclude extraneous data that is not relevant to the prediction [43,46]. GRU preserves LSTM's characteristics to handle vanishing gradients. GRU’s gates are given in sigmoid activations, thus guaranteeing their values fall within the range of (0, 1), which can be used for benign and malicious traffic classifications. Additionally, the cell c_t_ and hidden h_t_ state merge into one in the GRU, meaning that h_t_ = c_t_. Mathematical expressions describe the flow of information within the GRU layers represented as follows.


$$z_{t}=\sigma \left(u_{z}x_{t}+w_{z}h_{t-\text{1}}+b_{z}\right).$$ (8)

The update gate at time *t* is computed using Eq. (8), which involves multiplying the input by the weight.


$$r_{t}=\sigma \left(u_{r}x_{t}+w_{r}h_{t-\text{1}}+b_{r}\right).$$ (9)

Eq. (9) illustrates the calculation of the reset gate, with the update gate at time *t* executing the input multiplied by the weight.


$$\overset{\sim }{\underset{t}{h}}=\text{tanh}\left(u_{h}x_{t}+w_{h}\left(r_{t}⊗h_{t-\text{1}}\right)+b_{h}\right).$$ (10)

Eq. (10) describes the current memory, where a weight scales the input.


$$h_{t}=\left(\text{1}-z_{t}\right)⊗h_{t-\text{1}}+\overset{\sim }{\underset{t}{h}}⊗z_{t}.$$ (11)

Eq. (11) depicts the final memory of the time step, where element-wise multiplication is applied to the update gate.

Where u_z_, u_r_ w_z_ and w_r_ denote the weight matrices, b_z,_ and b_r_ represent the bias vectors.

#### 3.4.3. XGBoost.

XGBoost is a sophisticated algorithm that utilizes gradient tree boosting to handle complex machine learning tasks efficiently. XGBoost was initially presented by Chen et al [47] in 2016. It accumulates regularization to handle the weighted estimation of data quantiles and spares data for learning trees, then optimizes the loss function [48]. Additionally, it provides some information that aids in the development of a quick and tree-boosting technique. These insights include patterns for cache access, compression, and data sharding. In terms of accuracy and speed, the XGBoost method outperforms other ML algorithms for these strategies and insights [49].

In our work, we utilized eXtreme gradient Boosting as the meta-learner, leveraging its advantages. XGBoost adopts an additive learning scheme with second-order estimation, including the first-order derivative, which is known as the gradient, and the second-order derivative, which is known as the Hessian of the loss function. Both the Hessian and gradient of the loss function are defined in [43], as shown in Eqs. (12,13).


$$l^{p}=\sum_{i=i}^{n}[\;g_{i},\;f_{p}\;\left(x_{i}\right)+\frac{\text{1}}{\text{2}}\;h_{i\;}\left(f_{p}\;\left(x_{i}\right)\right)\text{2}]\;+\Omega (f_{p})$$ (12)

$$L^{p}=\sum_{i=\text{1}}^{n}l{(y}_{i},\overset{\left(p-\text{1}\right)}{\underset{i}{z}}+f_{p\;}(x_{i}))+\Omega (f_{p\;})$$ (13)

#### 3.4.4. Stacked ensemble approach.

**Fig 7:**
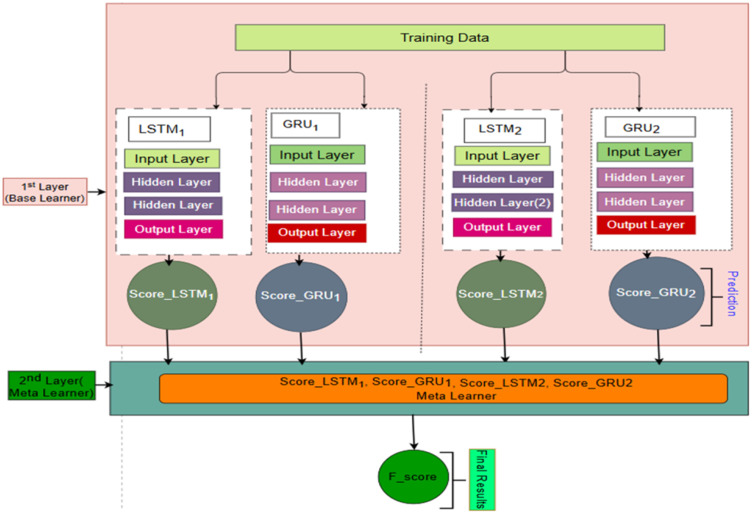
Framework of the Proposed stacked ensemble model.

Stacked ensemble learning focuses on combining multiple learning algorithms into a group, allowing them to work together to achieve better performance than using each algorithm individually. This ensemble learner has the potential to deliver improved performance. In our proposed stacked ensemble learning approach, the first layer comprises several base learners, including GRU and LSTM (LSTM1, GRU1, LSTM2, and GRU2), and XGBoost is employed as the meta-classifier in the second layer. The suggested framework is presented in Fig 7.

Unlike most ensemble methods that rely on a simple threshold-based voting mechanism, our approach focuses on learning the final output using the predictions of the base classifiers. First, the original training dataset is divided horizontally into k folds (in our study, k = 5), ensuring that all folds retain the same features and dimensional structure. Each fold contains an identical number of samples, is maintained by the Stratified K-Fold function. Subsequently, each base learner is trained on four of the training folds and then generates predictions for the remaining validation fold. The second meta-feature is created from predictions called out-of-fold predictions of each model, representing the base layer models’ results as Score_LSTM1, Score_GRU1, Score_LSTM2, and Score_GRU2. Lastly, stacked out-of-fold predictions from the base learner were used as meta-feature data for XGBoost. Our proposed stacked ensemble model as illustrated in Algorithm 2.

**Algorithm 2:** **Proposed Stacked ensemble learning model**

**Input:**

Dataset CIC and HKD

The numbers of recurrent neural network base classifiers, N

An ensemble classifier C

**Output:**

The trained stacked ensemble model

**Step 1: Data Preparation**

● Divide Dataset into number of k-fold

● Apply Stratified K-fold CV to the training dataset.

● Reshape input data for LSTM and GRU

**Step 2: Train Base Learners**

● Each classifier trained on the relevant k-1 fold and validated 1-fold.

● Generate prediction of each classifier (Score_LSTM_1_, Score_GRU_1_,

Score_LSTM_2_ and Score_GRU_2_)

**Step 3: Prepare Meta-Classifier Training Data**

● Create new Meta dataset from (Score_LSTM_1_, Score_GRU_1_,

Score_LSTM_2_ and Score_GRU_2_)

● Train the XGBoost classifier

Step 4: Model Testing

● Evaluate the proposed model's performance by testing it on the test dataset.

## 4. Results and discussion

In this section, we provide models, environment, and hyper-parameter optimization, as well as performance metrics used to measure the performance of the introduced models. In addition to this performance evaluation of sequential models, our proposed stacking learning approach and other models built for comparison are presented. Finally, we present a performance comparison of proposed stack model and existing previous works.

### 4.1. Experiment environment and parameters

Our research employed a sophisticated stacked ensemble approach, integrating predictions from various base models (GRU1, LSTM1, GRU2, LSTM2) and utilizing a meta-model (XGBoost) for the final classification. Initially, we trained the base models using stratified k-fold cross-validation, generating out-of-fold (OOF) predictions for the training data. These OOF predictions were consolidated into meta-features, serving as input features for the meta-model to train on. A meta-model was then trained on the complete set of OOF prediction meta-features alongside the corresponding true labels, allowing it to capitalize on the strengths of all base models while mitigating individual drawbacks.

For the testing phase, we fully trained the base models on the entire training dataset before using them to generate predictions for the unseen test set. These test set predictions from each base model were combined into a stacked prediction test feature set. The previously trained meta-model then processed these test set predictions to produce the final test set predictions, ensuring an unbiased assessment of the model's performance.

Our study also addressed class imbalance issues through the use of Stratified K-Fold, which maintained the original class distribution in all training, validation, and test folds. Additionally, we improved model performance through hyperparameter optimization. Testing all possible parameter combinations with a wide range of values demands significant time and resources. For the base learner, we mostly used default hyper-parameters while configuring the recurrent nodes, learning rate, and hidden nodes. Similarly, for the meta-learner (XGBoost), we optimized max_depth, learning_rate, and n_estimators using the same method, while keeping the rest at their default values, as detailed in Table 4. The dataset was divided into 80% for training and 20% for testing for model evaluation.

**Table 4:** Hyperparameters.

Parameters	Values	LSTM1	GRU1	LSTM2	GRU2	XGBoost
learning-rate	{10 ^− 3^, 10 ^− 4^, 10 ^− 5^, 10^−7^}	10 ^− 4^	10 ^− 4^	10 ^− 4^	10 ^− 4^	10 ^− 5^
dropout-rate	{0.2, 0.3, 0.5, 0.7, 0.8}	0.7	0.7	0.6	0.6	---
recurrent nodes	{20, 32, 40, 64}	32	32	64	64	---
hidden nodes	{16, 20, 32 50}	32	32	32	32	---
n_estimator	{50, 100, 500}	---	---	---	---	500
max depth	{3, 15, 1}	---	---	-----	---	3

### 4.2. Performance evaluation metrics

To analyze the model's performance, this study used four performance evaluation parameters, which are represented in Table 5.

**Table 5:** Performance Evaluation Parameters.

		Actual label	
		Malicious	Benign
Predicted label	Malicious	TP	FP
	Benign	FN	TN

Our evaluation metrics are largely based on specific parameters: True Positive (TP) is the count of malicious traffic accurately identified. False Positive (FP) is the number of benign traffic incorrectly classified as malicious, False Negative (FN) is the count of malicious traffic wrongly labeled as benign, and True Negative (TN) is the number of benign traffic correctly identified as benign. Using these parameters, we evaluated our model's performance through six key metrics: precision, accuracy, Recall, AUC-ROC, F1-score, and AUC-PR. These metrics are crucial for assessing the model's performance in detecting and classifying malicious DoH traffic, providing a detailed understanding of the model's reliability and precision in identifying malicious activities [50].

### 4.3. Result analysis and discussion

In this section, we present the results of our proposed model, single classifier, and other models, such as RF, DT, and MLP. To ensure fairness, all models were trained for an equal number of epochs. In our study, we evaluated performance of all models across CIC-DoH and HKD-DoH dataset. We first evaluated the performance of base learners, LSTM1, GRU1,LSTM2 and GRU2 to identify benign and malicious samples. As we can see from the results presented in Table 6 and Table 7 across both datasets, the sequential models GRU and LSTM perform very well. That's why we chose those models as base learners for classifying intrusion in encrypted DoH Network traffic and further used them to build a robust ensemble learning approach.

**Table 6:** Performance comparison of proposed model, single classifier and built models on CIC-DoH dataset.

Model	Accuracy	Precision	Recall	F1score	AUC-ROC	AUC-PR
GRU1	0.9767	0.9860	0.9890	0.9875	0.9825	0.9984
LSTM1	0.9847	0.9886	0.9949	0.9917	0.9937	0.9995
GRU2	0.9792	0.9882	0.9893	0.9888	0.9896	0.9989
LSTM2	0.9876	0.9909	0.9957	0.9933	0.9961	0.9997
MLP	0.9775	0.9965	0.9793	0.9878	0.9944	0.9996
RF	0.9832	0.9943	0.9876	0.9909	0.9965	0.9997
DT	0.9788	0.9950	0.9781	0.9884	0.9981	0.9998
**Ours**	**0.9965**	**0.9967**	**0.9996**	**0.9981**	**0.9999**	**1.0000**

**Table 7:** Performance comparison of the proposed model, single classifier, and built models on the HKD-DoH dataset.

Model	Accuracy	Precision	Recall	F1-score	AUC-ROC	AUC-PR
GRU1	0.9920	0.9920	0.9985	0.9953	0.9991	0.9998
LSTM1	0.9985	0.9987	0.9995	0.9991	0.9996	0.9999
GRU2	0.9662	0.9632	0.9979	0.9803	0.9865	0.9970
LSTM2	0.9787	0.9789	0.9962	0.9874	0.9862	0.9967
MLP	0.9822	0.9236	**1.0000**	0.9603	0.9823	0.9805
RF	0.9965	0.9859	0.9981	0.9920	1.0000	0.9999
DT	0.9881	0.9576	0.9886	0.9729	0.9949	0.9660
**Ours**	**0.9998**	**1.0000**	0.9998	**0.9999**	**1.0000**	**1.0000**

#### 4.3.1. Performance analysis of models on CIC-DoH dataset.

**Fig 8:**
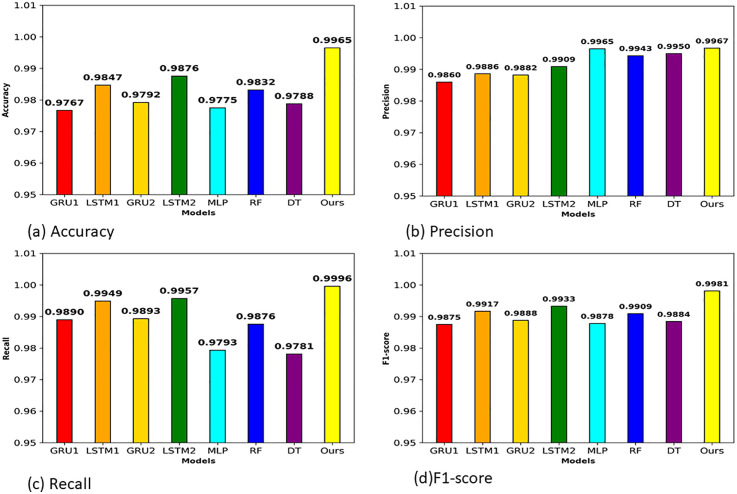
(a) Accuracy (b), Precision (c), Recall, and (d) F1-score of different models for the CIC-DoH dataset.

Table 6 and Fig 8 present performance evaluation of proposed stacking meta model and others models on CIC-DoH dataset. Our proposed stacked ensemble model shows strong performance across nearly all evaluation metrics. A proposed stacked ensemble approach scored accuracy of 0.9965,0.9967 of precision, recall of 0.9996, F1-score of 0.9981, 0.9999 AUC-ROC and 1.0000 perfect of AUC-PR. The experimental outcome confirmed that our stacking approach effectively combined strengths of four base learners and compensating weakness of individual classifiers. Among individual base classifiers LSTM1 indicates strong performance by scoring accuracy of 0.9847 and recall of 0.9949. This indicates that LSTM1 correctly classifies malicious DoH traffic while missing few actual malicious flows. Also, LSTM2 achieved 0.9876 of accuracy and recall of 0.9957, this may result from its deeper architecture and the more conservative learning rate applied to LSTM2, which helps capture subtle sequential patterns. On the other hand, GRU variants show strong performance but slightly lower performance. GRU1 classifier scored 0.9767 of accuracy and recall of 0.9890. While GRU2 achieves 0.9792 accuracy with recall of 0.9893. This minimal performance gap between both GRU and LSTM shows both models can handle this task very well, with the LSTM benefiting slightly from its gating structure when learning the temporal behavior of DoH flows. Regarding machine learning models RF, DT and MLP they achieved competitive results but slightly less than sequential models on most metrics. Random Forest scored 0.9832 of accuracy and notable precision of 0.9943, it produces very few false positive. Decision Tree reaches accuracy of 0.9788 but scored lowest recall of 0.9781 among all models.

Decision Tree reaches accuracy of 0.9788 but scored lowest recall of 0.9781 among all models. This indicates Decision Tree misclassify actual malicious traffic as benign traffic. MLP model achieved accuracy of 0.9775 with higher precision of 0.9965, yet its recall drops to 0.9793, showing a trade-off where the model becomes overly conservative in its malicious predictions. A notable observation can see from Fig (b) that our proposed stacking meta learning model achieved precision of 0.9967 while simultaneously scoring recall of 0.9996 as shown in Fig 8(c). This balanced performance of results is significant because recall and precision often show an opposite relationship, improving one comes at the cost of the other. The proposed stacked ensemble overcomes this trade off by learning from the complementary prediction patterns of four various base models through the XGBoost meta-learner. As we can also see from Fig(d), F1-score comparison further highlights this advantage, where proposed stacking model scored an F1-score of 0.9981, surpassing best individual model LSTM2 scored 0.9933 by a clear margin. Additionally, the AUC-ROC of 0.9999 and 1.0000 of AUC-PR presented in Table 6 demonstrate that the proposed model maintains discriminative ability across all classification thresholds, not only just at a single operating point.

**Fig 9:**
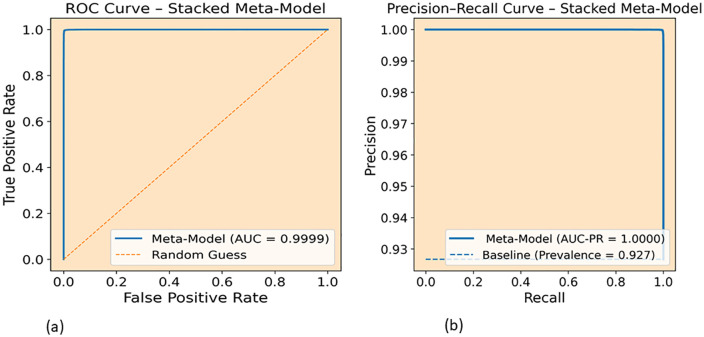
ROC and Precision-Recall curves of the proposed stacked meta-model on the CIC-DoH dataset: (a) ROC curve (AUC = 0.9999), (b) Precision-Recall curve (AUC-PR = 1.0000).

Furthermore, we visualized AUC-ROC and AUC-PR of proposed stacked meta-model on the CIC-DoH dataset on Fig 9. The ROC curve in Fig 9(a) presses tightly against the top-left corner with an AUC of 0.9999, and the clear gap from the diagonal random guess line shows the model can reliably separate malicious from benign DoH traffic across nearly all thresholds. In Fig 9(b), the Precision-Recall curve achieves a perfect AUC-PR of 1.0000, staying flat near the top even as recall increases, which means precision hardly drops when the model tries to catch more malicious. This matters especially because the CIC dataset carries a baseline prevalence of 0.927, a level of imbalance where most classifiers would see precision fall sharply at high recall. These two curves together confirm that the results presented in Table 6 hold consistently across all operating points rather than depending on a single favorable threshold

**Fig 10:**
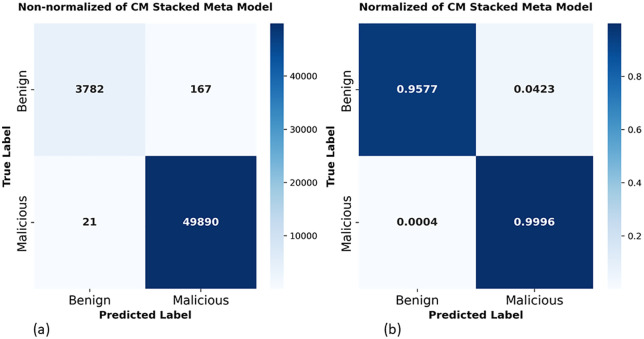
Confusion matrix of proposed stacking on CIC-DoH dataset: (a) non-normalized cm, (b) normalized cm.

In addition, the confusion matrix of our proposed stacked meta model on CIC dataset shown in Fig 10. Fig 10(a) presented non-normalized matrix raw sample counts across a four classification results. Out of 3,949 actual benign sample in test set, stacked meta model correctly classifies 3,782 as true negatives (benign) and misclassifies 167 as malicious. While on malicious side our model correctly detects 49,890 out of 49,911 actual malicious traffic with only 21 malicious traffic slipping through as benign (false negatives). These results show that the model achieves remarkable performance with total misclassification of just 188 samples out of 53,860. Fig 10(b) places these numbers into proportion and reveals per-class detection rates. A proposed stacked meta model correctly classifies 0.9577 of all benign traffic, meaning roughly 95.77% of benign flows are properly recognized, while 0.0423 are incorrectly flagged as malicious. For the malicious class, the model achieves 0.9996 recall, correctly identifying 99.96% of all malicious traffic and misclassifying a mere 0.0004 as benign. For network security, this is a sensible bias since missing a genuine attack carries far more risk than raising a false alarm. With only 21 missed malicious flows out of 49,911, the proposed framework delivers highly reliable detection on the CIC-DoH dataset.

#### 4.3.2. Performance analysis of models on HKD-DoH dataset.

**Fig 11:**
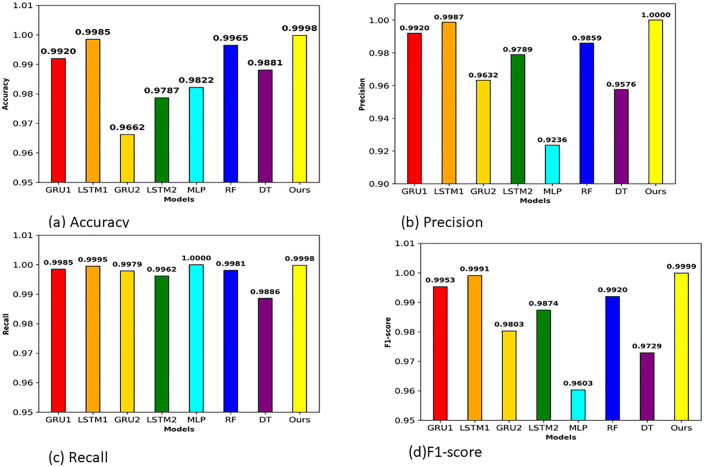
(a) Accuracy (b) Precision (c) Recall and (d) F1-score of different models for HKD-DoH dataset.

To ensure across dataset performance validation we also evaluated proposed stacking meta model, base learners and others models on the HKD-DoH dataset. As shown in Table 7 and Fig 11 proposed stacked ensemble model again also shows outstanding results by achieving 0.9998 of accuracy, 1.0000 of precision, F1-score of 0.9999, and perfect scores of 1.0000 for both AUC-ROC and AUC-PR. These results confirmed that the stacking framework maintains its effectiveness even when tested on newly emerging tunneling tools (tuns, tcp-over-dns, dnstt) used to generate the HKD dataset, which differ considerably from the tools covered in the CIC dataset.

Among base learners, LSTM1 demonstrate strong performance scoring 0.9985 of accuracy, 0.9987 precision, 0.9995 recall, and 0.9991 F1-score. Its AUC-ROC and AUC-PR values (0.9996 and 0.9999) sit remarkably close to those of the stacked model. GRU1, the second best-performing among individual models, its performance reaches 0.9920 accuracy and 0.9985 recall. GRU2 drops to 0.9662 accuracy and 0.9632 precision, and LSTM2 settles at 0.9787 accuracy with 0.9789 precision. These result differences are observed due to hyperparameter settings. Machine learning based models produce mixed outcomes on the HKD dataset. Random Forest reaches 0.9965 of accuracy with a perfect 1.0000 of AUC-ROC, showing strong performance. However, its precision of 0.9859 suggests it generates more false positives. Decision Tree achieves 0.9881 accuracy but records a lower precision of 0.9576 and the weakest F1-score of 0.9729 among all models. MLP achieved a perfect recall of 1.0000, meaning it detect every single malicious flow, yet its precision drops to 0.9236, the lowest across all models.

Furthermore, we visualize performance of models in Fig 11. Fig 11(a), shows the accuracy proposed stacking model reaches 0.9998, clearly above others models accuracy while LSTM1 and RF accuracy near close to it. Fig 11(b) demonstrates precision comparison of models, a proposed model achieved 1.0000, while MLP scored only just 0.9236, highlighting the massive difference in false positive rates between the two approaches. Fig 11(c) shows that recall values are generally high across most models, yet the proposed model still manages to secure 0.9998 without sacrificing precision, something no other model accomplishes on this dataset. F1-score of proposed stacking model clearly separates it from every other approach as shown in Fig 11(d) with score of 0.9999. When combined with the AUC-ROC and AUC-PR values of 1.0000 presented in Table 7, these results demonstrate that the stacked ensemble model achieves comprehensive and reliable classification performance across every threshold and metric on the HKD-DoH dataset.

**Fig 12:**
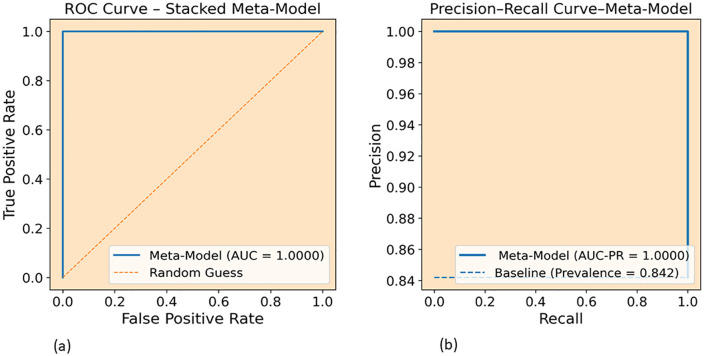
ROC and Precision-Recall curves of the proposed stacked meta-model on the HKD-DoH dataset: (a) ROC curve (AUC = 1.0000), (b) Precision-Recall curve (AUC-PR = 1.0000).

Additionally, Fig 12 shows the ROC and Precision-Recall curves of the proposed stacked meta-model on the HKD-DoH dataset. The ROC curve in Fig 12(a) reaches a perfect AUC of 1.0000, reaching the upper-left corner with no visible gap from the ideal boundary, meaning the model completely separates malicious and benign traffic at every threshold. The Precision-Recall curve in Fig 12(b) is equally strong with an AUC-PR of 1.0000, holding precision near the maximum across the full recall range. This is worth noting because the HKD dataset has a lower malicious class prevalence of 0.842 compared to 0.927 in CIC, which normally makes it harder to keep precision high. Achieving flawless scores on both curves while dealing with newer tunneling tools (tuns, tcp-over-dns, dnstt) that were absent from the CIC data supports the generalizability of the proposed framework and confirms that the results in Table 7 hold across all classification thresholds.

**Fig 13:**
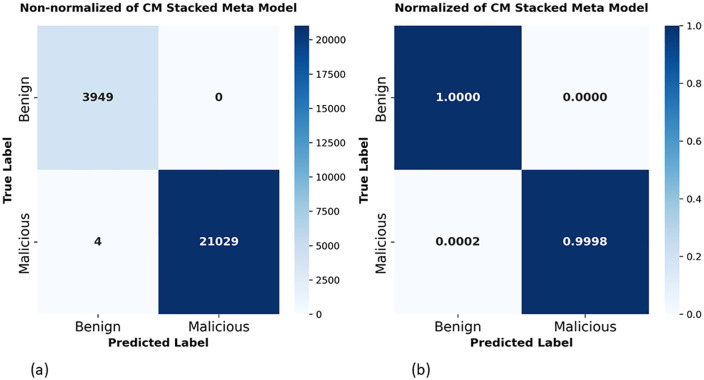
Confusion matrix of proposed stacking on HKD-DoH dataset: (a) non-normalized cm, (b) normalized cm.

Also, confusion matrix of the proposed stacked meta-model on the HKD-DoH dataset demonstrated in Fig 13. The non-normalized confusion matrix in Fig 13(a) shows that all 3,949 benign samples are correctly classified with zero false positives, while 21,029 out of 21,033 malicious traffic are correctly detected, misclassify only 4 false negatives. In total, just 4 samples out of 24,982 are misclassified. The normalized matrix in Fig 13(b) confirms these numbers in proportional terms: the benign class reaches a perfect 1.0000 correct rate with 0.0000 false positives, and the malicious class achieves 0.9998 recall with only 0.0002 going undetected. Compared to the CIC results Fig 10, where 167 false positives and 21 false negatives misclassified, the HKD results show clear improvement false positives droplet to zero and false negatives only just 4.

**(i). The truth behind the performance of a proposed stacked meta learner model:** This strong result comes from the diversity of four distinct base models with different neural architectures (GRU vs. LSTM) and contrasting hyperparameters, which were utilized with learning, dropout, recurrent nodes and different dense layers. This creates models that performs in different scenarios. Each base model develops unique strengths and blind spots, ensuring that when one model struggles with certain data patterns, others compensate effectively. Ensemble models developed in different scenarios enable the intrusion detection system to easily detect malicious activity in encrypted DoH networks. The other reason for a robust outcome is that the training methodology centers on cross-validation stacking, which prevents the meta-model from overfitting by ensuring it only sees out-of-fold predictions during training. This eliminates data leakage and creates genuinely unbiased performance. The XGBoost meta-learner doesn't just average predictions, it learns complex decision rules about when to trust each base model.

Generally, a robust system is created where diversity prevents systematic failures, proper validation ensures generalizability, and intelligent combination maximizes the collective intelligence of the ensemble. The result is a detection system that achieves near-perfect performance while maintaining reliability across different network conditions and attack scenarios.

#### 4.3.3. Performance comparison between the proposed stacked approach and existing works.

This section presents a comparison of the proposed stacked ensemble learning approach’s results with those of existing frameworks, which used CIC datasets to evaluate their models. Table 8 presents comparison of proposed stacked ensemble approach against existing methods on the CIC-DoH dataset. As shown in Table 8, CNN based models [12], performance varies considerably depending on the architecture. The 1D CNN-5 variant achieves the best results in that group with 0.9841of accuracy and 0.9884 of precision, while the 2D CNN-5 results the weakest scores of 0.9593 accuracy and recall of 0.9487, suggesting that two-dimensional convolutions are less effective at detecting the traffic patterns present in DoH traffic. The 1D CNN-4 and 2D CNN-6 models both scored accuracy of 0.9770 and 0.9768 respectively. however, CNN models [12], does not reported AUC-ROC or AUC-PR values, which limits a full comparison of their discriminative power across thresholds.

**Table 8:** Performance of comparison between the proposed approach and existing works on the CIC dataset.

Ref	Model	Accuracy	Precision	Recall	F1score	AUC-ROC	AUC-PR
[12]	1D CNN-4	0.9770	0.9762	0.9794	0.9770	NA	NA
	1D CNN-5	0.9841	0.9884	0.9765	0.9841	NA	NA
	2D CNN-5	0.9593	0.9609	0.9487	0.9592	NA	NA
	2D CNN-6	0.9768	0.9679	0.9837	0.9770	NA	NA
[7]	DoH-TriCGAN	0.9974	0.9978	0.9977	0.9976	0.9985	0.9996
[29]	Transformer	NA	0.994	0.994	0.994	NA	NA
	**Proposed approach**	**0.9965**	**0.9967**	**0.9996**	**0.9981**	**0.9999**	**1.0000**

The other existing models, DoH-TriCGAN [7], appear as the strong performer, achieving an 0.9974 accuracy, 0.9977 recall, 0.9978 precision, 0.9976 F1-score, AUC-PR and AUC-ROC values 0.9996 and 0.9985, respectively. These are strong numbers, yet the proposed approach still surpasses DoH-TriCGAN on recall (0.9996 vs. 0.9977), F1-score (0.9981 vs. 0.9976), AUC-ROC (0.9999 vs. 0.9985), and AUC-PR (1.0000 vs. 0.9996). The higher recall is particularly meaningful in a security context because it means the proposed model misses fewer actual malicious flows. The Transformer-based model presented in [29] scored 0.994 of precision, recall, and F1-score but does not provide accuracy, AUC-ROC, and AUC-PR results. Our proposed model clearly surpasses it on the available metrics.

Overall, the proposed stacked meta-model outperforms all compared existing models across nearly all metrics. While DoH-TriCGAN is slightly ahead on accuracy (0.9981 vs 0.9965) and precision (0.9978 vs 0.9967). However, proposed model compensates with a noticeably stronger recall and superior AUC scores, which reflect more reliable and consistent classification across all decision thresholds. These experimental results indicate that the stacking of diverse sequential base learners with an XGBoost meta learner offers a genuine performance advantage over CNN, Transformer and GAN-augmented approaches for malicious DoH traffic detection.

#### 4.3.4. Time complexity analysis.

To analyze the effectiveness and operational performance of our stacked solution, All experiments were performed on a workstation equipped with an Intel Core i5 CPU, and 32 GB RAM, using Python 3.12.7 and TensorFlow 2.14.0. Even though dramatic time improvements are achieved, as shown in Table 9, which demonstrates the superiority of our proposed stacked approach, where the XGBoost meta-model significantly outperforms individual RNN-based methods. As shown in Table 9, on CIC-DoH dataset individual base learners need more training time ranging from 107.81 seconds (LSTM1) to 121.20 seconds (LSTM2) demonstrating the computational cost of recurrent neural network processing sequential data across 5-folds stratified. The proposed meta model required very small training time only around 2.72 seconds and inference time is 0.2912 seconds, while base learners’ models need between 8.50 and 11.40 seconds to generate predictions.

**Table 9:** Time complexity analysis of models.

Dataset	time (s)	GRU1	LSTM1	GRU2	LSTM2	Proposed model
CIC-DoH	Training time	108.43	107.81	115.12	121.20	2.72
	Inference time	11.40	9.56	8.50	10.81	0.2912
HKD-DoH	Training time	53.42	55.70	36.48	39.93	1.26
	Inference time	2.80	3.58	1.71	1.94	0.1257

As we observed for Table 9 training time of base models decrease to 36.48–55.70 seconds range on HKD-DoH dataset, this is due to the smaller sample size of HKD-DoH dataset. A proposed meta model again just finished training in 1.26 seconds and inference in 0.1257 seconds. This efficiency results from the fact that the XGBoost meta-learner approaches operates on only four pre-computed prediction scores from the base models rather than the full original feature set, which drastically reduces the input dimensionality and computational load. These results confirm that the stacking approach not only improves detection accuracy but also adds minimal overhead at deployment time, making it well suited for real-time intrusion detection in high-speed network environments where fast response is essential.

## 5. Conclusion

In this study, we considered the security issues of encrypted DoH networks and proposed a robust ensemble meta learning approach to detect intrusions in DoH Network Traffic. Our robust method, developed from the diversity of four base models (two GRUs and two LSTMs), was designed to handle different scenarios varying in learning rates, dropout levels, and architectures, and utilized XGBoost as a meta-learner model. In addition to stacking the diversity of base learners and training XGBoost on out-of-fold (OOF) predictions, we integrated feature selection using RFE with XGBoost and reduced the feature space from 29 to 13 features on the CIC dataset and from 29 to 20 on HKD dataset. Our experiments demonstrate that the proposed robust ensemble meta approach achieved remarkable performance on both datasets. On the CIC-DoH dataset (Table 6 and Fig 8) proposed model achieved recall of 0.9996, 0.9981 F1-score, AUC-ROC of 0.9999 and 1.0000 AUC-PR. On the HKD-dataset the proposed ensemble meta-approach performs reached precision of 1.0000, 0.9998 of recall, 0.9999 of F1 score and 1.0000 for both AUC-ROC and AUC-PR. The ROC and Precision-Recall curves (Figs. 9 and 11) confirmed that these scores hold across all thresholds. Compared to existing models, the proposed approach surpassed prior approaches, including DoH-TriCGAN which scored a 0.9976 F1-score. In terms of effectiveness and operational feasibility in terms of time and resources, the proposed model is productive, while the XGBoost meta-model requires only 2.72 seconds for training and 0.29 seconds for inference on CIC-DoH dataset.

Furthermore, we would like to highlight some of the study's limitations. As the proposed approach is developed on a real tabular dataset collected for specific attacker samples, we are concerned about the performance of our model when it is deployed in real-time networks, where diverse types of attackers try to invade the network with sophisticated techniques. Our future work includes expanding our investigation by employing advanced sequential model architectures and adapting them to address various DoH security attack challenges, such as DGA. By implementing these sophisticated techniques, we aim to contribute to the development of more potent strategies for combating the multifaceted and evolving security threats within DoH systems.
